# Ectopic overexpression of LAPTM5 results in lysosomal targeting and induces Mcl-1 down-regulation, Bak activation, and mitochondria-dependent apoptosis in human HeLa cells

**DOI:** 10.1371/journal.pone.0176544

**Published:** 2017-05-02

**Authors:** Do Youn Jun, Hyejin Kim, Won Young Jang, Ji Young Lee, Kiyoshi Fukui, Young Ho Kim

**Affiliations:** 1 Laboratory of Immunobiology, School of Life Sciences and Biotechnology, College of Natural Sciences, Kyungpook National University, Daegu, Korea; 2 Institute of Life Science and Biotechnology, Kyungpook National University, Daegu, Korea; 3 Institute for Enzyme Research, Division of Gene Regulatorics, University of Tokushima, Kuramoto-cho, Tokushima, Japan; Columbia University, UNITED STATES

## Abstract

Human lysosomal-associated protein multispanning membrane 5 (LAPTM5) was identified by an ordered differential display-polymerase chain reaction (ODD-PCR) as an up-regulated cDNA fragment during 12-*O*-tetradecanoylphorbol 13-acetate (TPA)-induced differentiation of U937 cells into monocytes/macrophages. After TPA-treatment, the levels of *LAPTM5* mRNA and protein increased and reached a maximum at 18–36 h. In healthy human tissues, LAPTM5 mRNA was expressed at high levels in hematopoietic cells and tissues, at low levels in the lung and fetal liver, and was not detected in other non-hematopoietic tissues. *LAPTM5* mRNA was detected in immature malignant cells of myeloid lineage, such as K562, HL-60, U937, and THP-1 cells, and in unstimulated peripheral T cells, but was absent or barely detectable in lymphoid malignant or non-hematopoietic malignant cells. The LAPTM5 level in HL-60 cells increased more significantly during TPA-induced monocyte/macrophage differentiation than during DMSO-induced granulocyte differentiation. Ectopic expression of GFP-LAPTM5 or LAPTM5 in HeLa cells exhibited the localization of LAPTM5 to the lysosome. In HeLa cells overexpressing LAPTM5, the Mcl-1 and Bid levels declined markedly and apoptosis was induced via Bak activation, Δψm loss, activation of caspase-9, -8 and -3, and PARP degradation without accompanying necrosis. However, these LAPTM5-induced apoptotic events except for the decline of Bid level were completely abrogated by concomitant overexpression of Mcl-1. The pan-caspase inhibitor (z-VAD-fmk) could suppress the LAPTM5-induced apoptotic sub-G_1_ peak by ~40% but failed to block the induced Δψm loss, whereas the broad-range inhibitor of cathepsins (Cathepsin Inhibitor I) could suppress the LAPTM5-induced apoptotic sub-G_1_ peak and Δψm loss, by ~22% and ~23%, respectively, suggesting that the LAPTM5-mediated Δψm loss was exerted at least in part in a cathepsin-dependent manner. Together, these results demonstrate that ectopic overexpression of LAPTM5 in HeLa cells induced apoptosis via cleavage of Mcl-1 and Bid by a LAPTM5-associated lysosomal pathway, and subsequent activation of the mitochondria-dependent caspase cascade.

## Introduction

Lysosomal-associated multispanning membrane protein (LAPTM5), which is preferentially expressed in hematopoietic cells and localized to the lysosome, was initially isolated by a subtractive hybridization strategy between hematopoietic and non-hematopoietic cells [1]. LAPTM5 contains five hydrophobic transmembrane domains, with C-terminal tyrosine-based lysosomal targeting motifs [2]. In rat cerebellar cell culture, LAPTM5 in microglia is up-regulated in response to degeneration and apoptotic cell death of granule neurons, indicating the possible involvement of LAPTM5 in microglial activation and enhancement in phagocytosis toward dead neurons [3]. In rheumatoid arthritis, LAPTM5 is co-expressed with several known genes, which are expressed at low levels in resting macrophages and up-regulated during macrophage activation [4]. A recent study shows that LAPTM5 is a positive regulator of proinflammatory signaling pathways via facilitating NF-κB and MAPK signaling, and proinflammatory cytokine production in macrophages [5]. Since lysosomes are essential in the processing of foreign antigens by professional antigen-presenting cells and digestion of ingested materials in phagocytes, LAPTM5 might be associated with the proteolytic activity of lysosomes required for phagocytosis and antigen processing, and it may augment the inflammatory response in myeloid lineage immune cells.

Yeast two-hybrid analysis reveals that LAPTM5 is an interacting partner of Smurf2, an E3-ubiquitin ligase associated with the degradation of TGFβ signaling components that include the TGFβ receptor and Smad proteins, in human hepatocellular carcinoma HepG2 cells [6, 7]; the expression of *LAPTM5* mRNA increased 20-fold in HepG2 cells following TGFβ treatment. Further analysis using LAPTM5 as the bait identified several LAPTM5 partners, including ubiquitin, other E3 ubiquitin ligases, and proteins involved in endocytosis [7]. These results indicate that the role of LAPTM5 in lysosomal proteolysis can be extended to non-hematopoietic cells, and suggest that LAPTM5 might be a lysosomal transporter protein involved in the uptake of cellular proteins by the lysosome and may mediate their degradation. Recent studies using LAPTM5-deficient mice demonstrated that LAPTM5 is essential for lysosomal degradation of T cell and B cell receptors and thus contributes to suppression of the cell surface receptor-mediated activation of T and B cells [8, 9].

Besides the five membrane-spanning segments, LAPTM5 has three PY motifs (L/PPxY), which bind the WW domains of the Nedd4 family of ubiquitin ligases, and a ubiquitin interacting motif (UIM) in the C-terminus oriented toward the cytoplasmic side [9, 10]. The interaction of the PY motif of LAPTM5 and the WW domain of NEDD4-1, a HECT-type E3 ligase that belongs to the Nedd4 family, has been shown to be critical for the transport of LAPTM5-positive vesicles from the Golgi to the lysosome [10, 11]. Therefore, the specific interaction between the functional motifs of LAPTM5 and target proteins mediates the targeting of LAPTM5 to the lysosome and the role of LAPTM5 in lysosomal degradation of target proteins.

In relation to the involvement of LAPTM5 in neoplastic transformation, the inactivation of the LAPTM5 gene by chromosome rearrangement and DNA methylation is observed in human multiple myeloma cell lines [12]. Interestingly, the accumulation of LAPTM5-positive vesicles is associated with programmed cell death occurring during the spontaneous regression of neuroblastomas [13]. The cell death induced in neuroblastoma cells after Adenovirus-*LAPTM5* (Ad-*LAPTM5*) infection is not blocked by the pan-caspase inhibitor z-VAD-fmk, but is enhanced in the presence of both proteasomal and lysosomal inhibitors, causing the accumulation of LAPTM5-positive vesicles. Thus, it has been suggested that the loss of LAPTM5 can be associated with tumor progression by suppressing cell death; however, the biochemical mechanism underlying LAPTM5-mediated cell death remains unknown. Furthermore, it remains obscure whether the cell death caused by the LAPTM5 accumulation in neuroblastoma cells can be extended to cervical cancer HeLa cells.

Previously, as an attempt to isolate significantly up-regulated or down-regulated genes during 12-*O*-tetradecanoylphorbol 13-acetate (TPA)-induced terminal differentiation of U937 cells into monocytes/macrophages, we performed an ordered differential display-polymerase chain reaction (ODD-PCR), a method of displaying the 3'-end *Rsa*I-restriction fragments of cDNA [14]. As a result, we could identify the human apolipoprotein C2 gene (*APOC2*) which exhibits a significant enhancement in expression during TPA-induced differentiation of U937 cells [15]. In the present study, by further cloning and sequencing of the resulting cDNA clones selected from the ODD-PCR, we found that one 113-bp cDNA clone that was significantly up-regulated during the TPA-induced differentiation of U937 cells was the *LAPTM5* gene, encoding an integral lysosomal membrane protein. The level of *LAPTM5* mRNA differed in a cell type- and tissue type-specific, as well as a malignancy-related manner. The ectopic overexpression of LAPTM5 in human cervical epithelioid carcinoma HeLa cells resulted in lysosomal targeting of LAPTM5, a significant reduction in cell proliferation, and induction of apoptotic cell death, via mitochondrial damage and caspase cascade activation, without accompanying necrosis. The data demonstrate that LAPTM5, which was previously required for the pro-inflammatory signaling pathway in macrophages [5] and down-regulation of T cell and B cell receptors [8, 9], is associated with a lysosomal alliance to a mitochondrial apoptotic pathway by reducing the anti-apoptotic Mcl-1 protein level when ectopically overexpressed in HeLa cells.

## Materials and methods

### Kits, oligonucleotide primers, enzymes, reagents, bacterial strains, media, antibodies, and cells

The SuperScrip^TM^ system for cDNA synthesis was purchased from Life Technologies (Gaithersburg, MD, USA). All restriction enzymes and DNA-modifying enzymes including T4 DNA ligase, RNase, and T4 polynucleotide kinase were purchased from Boehringer Mannheim Corp. (Indianapolis, IN, USA). An Omnibase DNA sequencing kit, Taq DNA polymerase, and pGEM-T Easy Vector System I were purchased from Promega (Madison, WI, USA). Human multiple-tissue northern blot panels I and II, human immune system multiple-tissue northern blot panel II, and ExpressHyb hybridization solution for northern blot analysis were obtained from Clontech Laboratories, Inc. (Palo Alto, CA, USA). 12-*O*-tetradecanoylphorbol 13-acetate (TPA), 3,3'-dihexyloxacarbocyanine iodide (DiOC_6_), 3-(4,5-dimethylthiazol-2-yl)-2,5-diphenyltetrazolium bromide (MTT), 4',6-diamidino-2-phenylindole (DAPI), propidium iodide (PI), and the cathepsin D inhibitor (Pepstatin A) were obtained from Sigma-Aldrich (St. Louis, MO, USA). The broad range inhibitor of cathepsins (Cathepsin Inhibitor I) was obtained from Calbiochem (San Diego, CA, USA), and the pan-caspase inhibitor z-VAD-fmk was obtained from BD Biosciences (Chicago, IL, USA). Radioactive materials such as [γ-^32^P]ATP (~3,000 Ci/mmol), [α-^32^P]dCTP (~3,000 Ci/mmol) and [α-^35^S]dATP (~1,000 Ci/mmol), TaqStart antibody for PCR amplification, and the random primer labeling kit were obtained from Amersham (Arlington Heights, IL, USA). The nylon membrane (GeneScreen Plus) and [^3^H]TdR (2 Ci/mmol) were purchased from NEN Biotechnology System (Boston, MA, USA). Green fluorescence protein (GFP) vector (pFP-N1) was obtained from Clontech. *Escherichia coli* JM 109 used as the host strain for cDNA cloning was obtained from Promega. Bacterial media components were obtained from Difco Laboratories Inc. (Detroit, MI, USA). Rabbit antiserum produced against recombinant human LAPTM5 protein that was expressed using the *E*. *coli* system was prepared as previously described [16]. The anti-caspase-3 antibody was purchased from Pharmingen (San Diego, CA, USA), and the anti-poly (ADP-ribose) polymerase (PARP), anti-Bak, anti-Bax, anti-Bid, anti-Bcl-2, anti-Bcl-xL, anti-COX-1, anti-COX-2, anti-NF-κB p65, and anti-Mcl-1 antibodies were purchased from Santa Cruz Biotechnology (Santa Cruz, CA, USA). The anti-caspase-9, anti-caspase-8, and anti-IκBα antibodies were purchased from Cell Signaling Technology (Beverly, MA, USA), and the anti-Bak (Ab-1) antibody was obtained from Calbiochem. The anti-APOC2 antibody was purchased from Abcam (Cambridge, UK), and the anti-p47phox antibody was purchased from Assay Biotech (Sunnyvale, CA, USA). The anti-GAPDH antibody was purchased from Thermo Scientific (Rockford, IL, USA). Jurkat, Molt-3, K562, HL-60, THP-1, U937, Sup-T1, COLO 320DM, and HeLa cells were purchased from ATCC (Manassas, VA, USA). Human peripheral T-cells were prepared using heparinized blood obtained from healthy laboratory personnel by venipuncture as previously described [15]. This protocol was approved by the Ethics Committee of Kyungpook National University, Daegu, Korea. Informed written consent was obtained from the participant. Human leukemias (Jurkat, Molt-3, K562, HL-60, THP-1, and U937), lymphoma (Sup-T1), and COLO 320DM cells were maintained in RPMI 1640 (Hyclone, Gaithersburg, MD, USA) containing 10% FBS, 20 mM HEPES (pH 7.0), 5 × 10^−5^ M β-mercaptoethanol, and 100 μg/ml gentamycin. HeLa cells were maintained in DMEM (Hyclone) supplemented with 10% FBS, 20 mM HEPES (pH 7.0), 1 mM sodium pyruvate, 5 × 10^−5^ M 2-mercaoptoethanol, and 100 μg/ml gentamycin.

### Induction of differentiation of U937 and HL-60 cells

Terminal differentiation of U937 cells into monocytes/macrophages was induced by 32 nM TPA for 48 h as previously described [15, 17]. Differentiation of HL-60 cells into monocytes/macrophages was induced by 32 nM TPA for 60 h, whereas differentiation of HL-60 cells into granulocytes was induced by1.25% DMSO for 60 h [18]. To investigate growth arrest of cells during induced differentiation, [^3^H]TdR-incorporation into the DNA of TPA- or DMSO-treated cells was measured. Cells (5 × 10^4^ cells/well) were incubated with 32 nM TPA or 1.25% DMSO in a 96-well plate and pulsed for 4 h with 1 μCi of [^3^H]TdR at the times indicated. The cells were harvested and assayed by liquid scintillation to measure the incorporation of [^3^H]TdR.

### Flow cytometric analyses

Flow cytometric analyses of the cell cycle in TPA-treated U937 cells were carried out as previously described [15, 19]. The extent of necrosis was determined using a FITC-Annexin V apoptosis kit (Clontech, Takara Bio Inc., Shiga, Japan) as previously described [20]. The changes in the mitochondrial membrane potential (Δψm) were measured after staining with DiOC_6_ as described elsewhere [21]. The Bak activation was measured using an anti-Bak (Ab-1) antibody-specific for the conformationally active Bak protein as previously described [22].

#### ODD-PCR, DNA sequence analyses, and homology search

ODD-PCR was carried out as previously described [14, 15]. Total RNAs extracted from control and TPA-treated (18 h and 48 h) U937 cells were reverse transcribed with the T-primer 5'-GCGAGTCGACCG(T)_13_ using the SuperScript System. The synthesized double-stranded cDNA samples were digested by *Rsa*I, and then used for ligation with a pseudo-double-stranded adaptor (a long oligonucleotide, 5'-GCGTGAAGACGACAGAAAGGGCGTGGTGCGGAGGGCGGT; and a short oligonucleotide, 5'-ACCGCCCTCCGC). The ligation mixture was used for PCR amplification with an adaptor-specific primer (5'-TGTAGCGTGAAGACGACAGAA) and the T-primer. The PCR product was used for the amplification of the simplified 3'-end cDNA subsets. Individual AdE-primers (Adaptor-specific Extended; 5'-AGGGCGTGGTGCGGAGGGCGGTCCNN, where NN is GC or AG) required for the amplification were radiolabeled with [γ-^32^P]ATP using T4 polynucleotide kinase. After the ^32^P-labelled AdE-primer was added to a PCR mixture containing 250 μM dNTPs, 2.5 U of Taq DNA polymerase mixed with TaqStart antibody, 0.2 μM non-labeled TE-primer (T-Extended; 5'-GCGAGTCGACCG(T)_13_NN, where NN is AG, GG, GA, GT or GC), and 1 ng of the representative 3'-end cDNA fragment sample under investigation, the amplification reaction was performed with 23 cycles of 95°C for 30 s, 69°C for 30 s, and 72°C for 90 s.

The PCR products were subjected to electrophoresis on a 6% polyacrylamide sequencing gel. Autoradiographs were visually examined after the dried gel was exposed to X-ray film at -80°C. Individual DNA bands of interest on the dried gel were eluted into 20 μl of TE buffer (pH 8.0) at 70°C for 2 h. After the eluent was reamplified with the T-primer 5'-GCGAGTCGACCG(T)_13_ and non-extended adaptor-specific primer (5'-AGGGCGTGGTGCGGAGGGCGGT) for 20 cycles, the amplified product was electrophoresed on a 2% agarose gel and purified from the gel using the GeneClean II kit (Bio 101 Inc., Vista, CA, USA). The purified DNA fragment was cloned using a pGEM-T Easy Vector System I and sequenced using the Omnibase DNA cycle sequencing system. The DNA sequence was compared with reference sequences in the GenBank databases using the BLAST program.

### Preparation of cell lysates and western blot analyses

Cells were suspended in the lysis buffer (137 mM NaCl, 15 mM EGTA, 1 mM sodium orthovanadate, 15 mM MgCl_2_, 25 mM MOPS, 1 mM PMSF, 5.0 μg/ml proteinase inhibitor E-64, and 0.1% Triton X-100, pH 7.2), disrupted by sonication, and extracted at 4°C for 30 min. After centrifugation at 16,000 × g for 20 min, the supernatant was collected. Protein quantitation was performed using a Micro BCA kit (Pierce, Rockford, IL, USA). An equivalent amount of protein lysate (20 μg) was electrophoresed on 4%-12% NuPAGE gradient gels (Invitrogen/Novex, Carlsbad, CA, USA) with MOPS buffer, and then electrotransferred to Immobilon-P membranes (Millipore Corporation, Bedford, MA, USA). Specific antibody-mediated detection of proteins on the membrane was performed using an ECL Western blotting kit (Amersham, Heights, IL, USA) as previously described [20]. Densitometry was performed using ImageQuant TL software (Amersham, Arlington Heights, IL, USA). Arbitrary densitometric units for the protein of interest were normalized to the densitometric units of GAPDH.

### Northern blot analyses

Total RNA was extracted and isolated by solubilization in guanidine thiocyanate as previously described [19]. Equivalent amounts of total RNA (15 μg) were electrophoresed on 1% formaldehyde-agarose gels and transferred to nylon membranes. The nylon membrane was hybridized in ExpressHyb solution at 68°C for 2 h with a cDNA probe radiolabeled with [α-^32^P]dCTP, using the random primer labeling kit (Amersham), and washed according to the manufacturer's instructions.

### Transfection of HeLa cells, MTT assay, immunostaining, and fluorescence microscopy

HeLa cells were transfected with individual plasmid DNA constructs using Effectene Transfection reagent (Qiagen, Valencia, CA, USA) according to the manufacturer’s instructions. The proliferation and cell cycle distribution of HeLa cells transfected with pCAGGS, pcDNA3.1, pCAGGS-*LAPTM5*, or pcDNA3.1-*Mcl-1* were analyzed by MTT assay reflecting cell viability and flow cytometry, respectively, as previously described [19, 20]. To observe localization of GFP and GFP-LAPTM5 under a fluorescence microscope, HeLa cells transfected with a *GFP* vector or a *GFP*-*LAPTM5* vector were fixed with 4% paraformaldehyde for 30 min. Immunostaining of HeLa cells transfected with pCAGGS or pCAGGS-*LAPTM5* was performed, as previously described [22]. For nuclei detection the cells were stained with 100 μM DAPI solution for 10 min. Images were collected at × 200 magnification using an LSM 700 confocal laser scanning microscope (Carl Zeiss MicroImaging GmbH, Jena, Germany).

### Statistical analyses

Unless otherwise indicated, each result in this paper is representative of at least three separate experiments. Statistical analyses were performed using the Students t-test to evaluate the significance of differences between two groups and one-way ANOVA for between three or more than three groups. In all graphs, * indicates *p* < 0.05 between the untreated and treated cells. All data are expressed as the mean ± standard deviation (SD, for each group n≥3). One-way ANOVA followed by Dunnett’s multiple comparison test was also used for statistical analysis using the SPSS Statistics version 23 (IBM, Armonk, NY, USA).

## Results

### Identification of differentially expressed transcripts during TPA-induced differentiation of U937 cells

**Fig 1:**
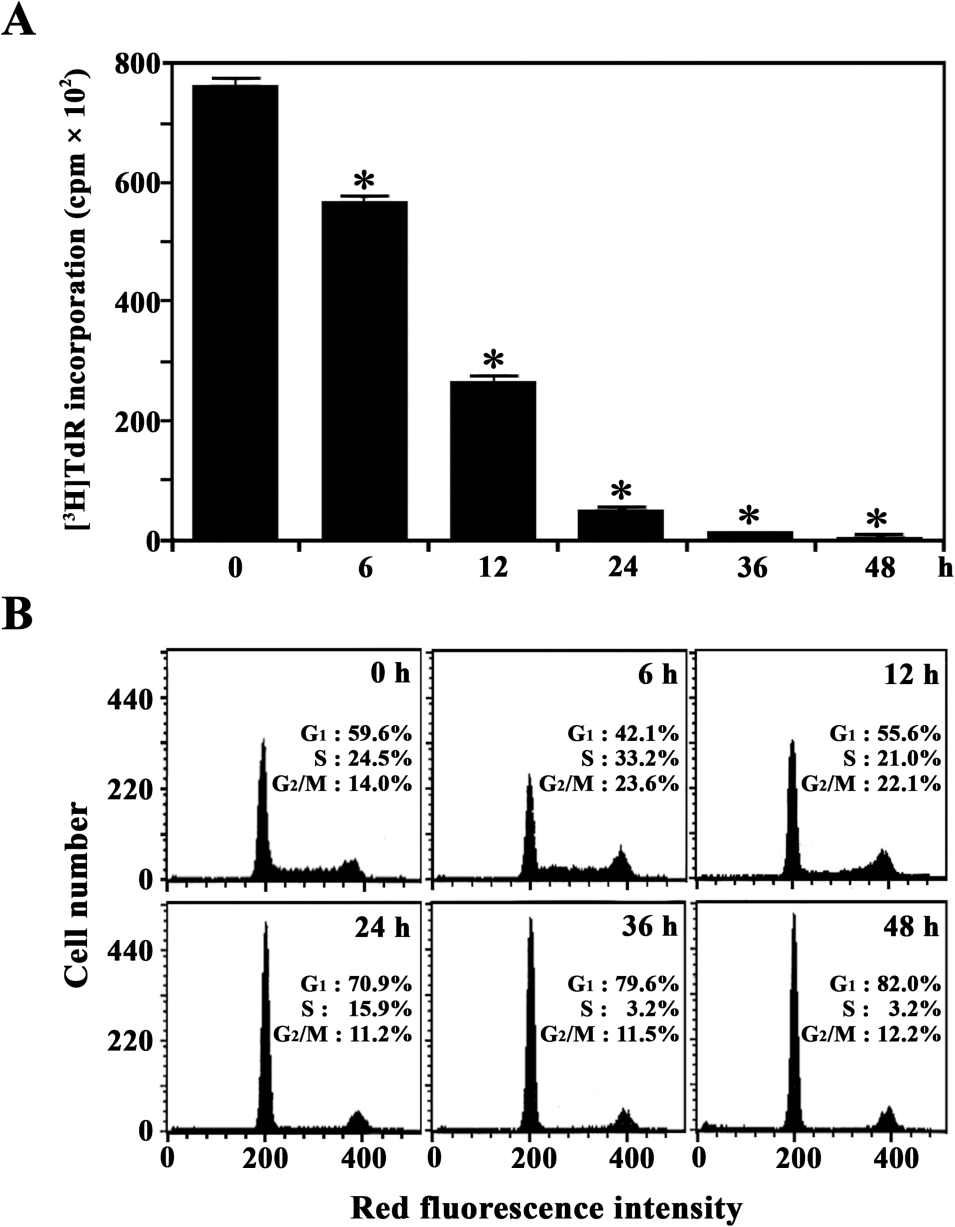
**Kinetic analysis of [**^**3**^**H]TdR-incorporation (A) and cell cycle distribution (B) during TPA-induced differentiation of U937 cells.** For the proliferation assay, U937 cells (5 × 10^4^ cells/well) were treated with 32 nM TPA in 96-well plates and pulsed for 4 h with 1 μCi of [^3^H]TdR at the indicated times. Each value is expressed as the mean ± SD (n = 3 with three replicates per independent experiment). *p < 0.05 compared to the control. After equivalent cultures (2.0 × 10^5^ cells/mL) were incubated, cells were harvested at the indicated times and stained with PI for flow cytometry.

We used ODD-PCR to investigate the gene expression profiles of U937 cells during TPA-induced differentiation into monocytes/macrophages and to identify uncharacterized genes. Because TPA-treated U937 cells are known to stop proliferation and differentiate into monocytes/macrophages, we measured the change in both the proliferative capacity and cell cycle distribution of U937 cells during induced differentiation. As shown in **Fig 1A**, U937 cells appeared to stop incorporating [^3^H]TdR 36 h after TPA treatment. Additionally, 36 h after TPA treatment, there was a significant enhancement in the proportion of cells arrested in the G_1_ phase, with approximately 79.6% of the cells in G_1_, 3.2% remaining in the S phase, and 11.5% in the G_2_/M phase (**Fig 1B**). By contrast, results from exponentially growing untreated U937 cells showed 59.6% of the cells in G_1_, 24.5% in the S phase, and 14.0% in the G_2_/M phase. These results indicate that treatment with 32 nM TPA caused growth arrest and induced differentiation of U937 cells into the terminal stage. Under these conditions, ODD-PCR was performed using the total RNA extracted from the exponentially growing U937 cells and U937 cells treated with 32 nM TPA for 18 h or 48 h. As a result, many bands representing PCR-amplified 3'-end *Rsa*I-restriction fragments of cDNAs exhibited different patterns of abundance (**S1 Fig**). When various PCR-amplified DNA fragments with altered expression levels were eluted from the gel, cloned, and sequenced, one of 113-bp cDNA clone, which appeared to be significantly up-regulated during TPA-induced differentiation of U937 cells, exhibited 100% similarity with the 3’-end of human *LAPTM5* gene (GenBank U51239). This nucleotide sequence was submitted to the GenBank under the accession number AF401210.

### Expression of *LAPTM5* mRNA and protein during differentiation of U937 cells

**Fig 2:**
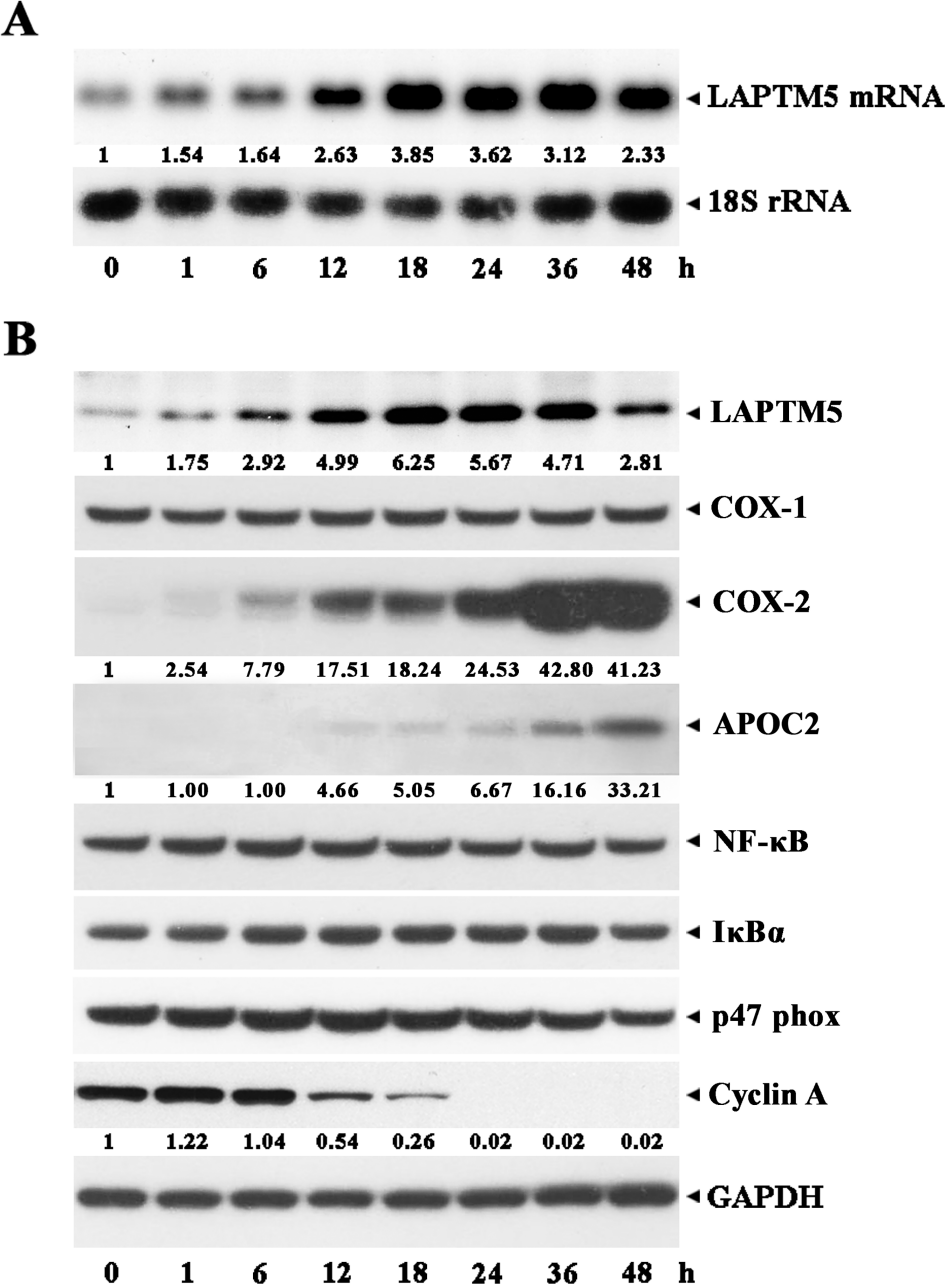
**Kinetic analysis of the expression of LAPTM5 mRNA (A) and LAPTM5 protein (B) during TPA-induced differentiation of U937 cells**. U937 cells were treated with 32 nM TPA for the indicated time and then total RNA and cell lysates were extracted. For northern blot analysis, total RNA (10 μg) were electrophoresed, transferred, and probed with ^32^P-labeled human LAPTM5, integrin α6 subunit, and 18S rRNA cDNA. After individual cell lysates were prepared, western blot analyses using anti-human LAPTM5, anti-COX-1, anti-COX-2, anti-APOC2, anti-NF-κB, anti-IκBα, anti-p47 phox, anti-cyclin A, and anti-GAPDH antibodies were performed as described in the Materials and Methods. A representative study is shown; two additional experiments yielded similar results.

We performed a northern blot analysis to quantify the expression of *LAPTM5* mRNA in TPA-treated U937 cells during induced differentiation into monocytes/macrophages. As shown in **Fig 2A**, the expression of the 2.4 kb *LAPTM5* mRNA, which is the only transcript detectable in the exponentially growing U937 cells, began to increase 1 h after treatment with TPA and reached a maximum level between 18 h and 36 h. Under these conditions, western blot analysis showed that the LAPTM5 protein level also significantly increased in accordance with the level of *LAPTM5* mRNA (**Fig 2B**). The level of cyclooxygenase-2 (COX-2), which is known to convert the arachidonic acid to an inflammatory mediator prostaglandin [23], was barely detected in untreated U937 cells and its level began to increase at 6 h and reached a maximum at 36–48 h after TPA treatment, whereas the cyclooxygenase-1 (COX-1) was detected in U937 cells at a constant level regardless of TPA treatment. The apolipoprotein C2 (APOC2) level was elevated and reached a maximum at 24–48 h after TPA treatment, as previously described [15]. The levels of transcription factor NF-κB and its inhibitor IκBα, and p47phox that is a cytosolic component of the NADPH oxidase complex [24], remained relatively constant. In contrast, the level of cyclin, which accumulates in the S phase of the cell cycle and supports cellular replication [25], decreased and became undetectable at 24 h, coinciding with when the cells barely or no longer incorporated [^3^H]TdR.

Following cell growth arrest, up-regulation of the COX-2 and APOC2 protein levels, and down-regulation of cyclin A protein level, all of which were previously observed along with TPA-induced differentiation of U937 cells into monocytes/macrophages [15, 17, 23, 25, 26], were accompanied by the upregulation of LAPTM5. These results demonstrate that the LAPTM5 level significantly increased during TPA-induced differentiation of U937 into monocytes/macrophages.

### Cell and tissue distribution of *LAPTM5* mRNA

**Fig 3:**
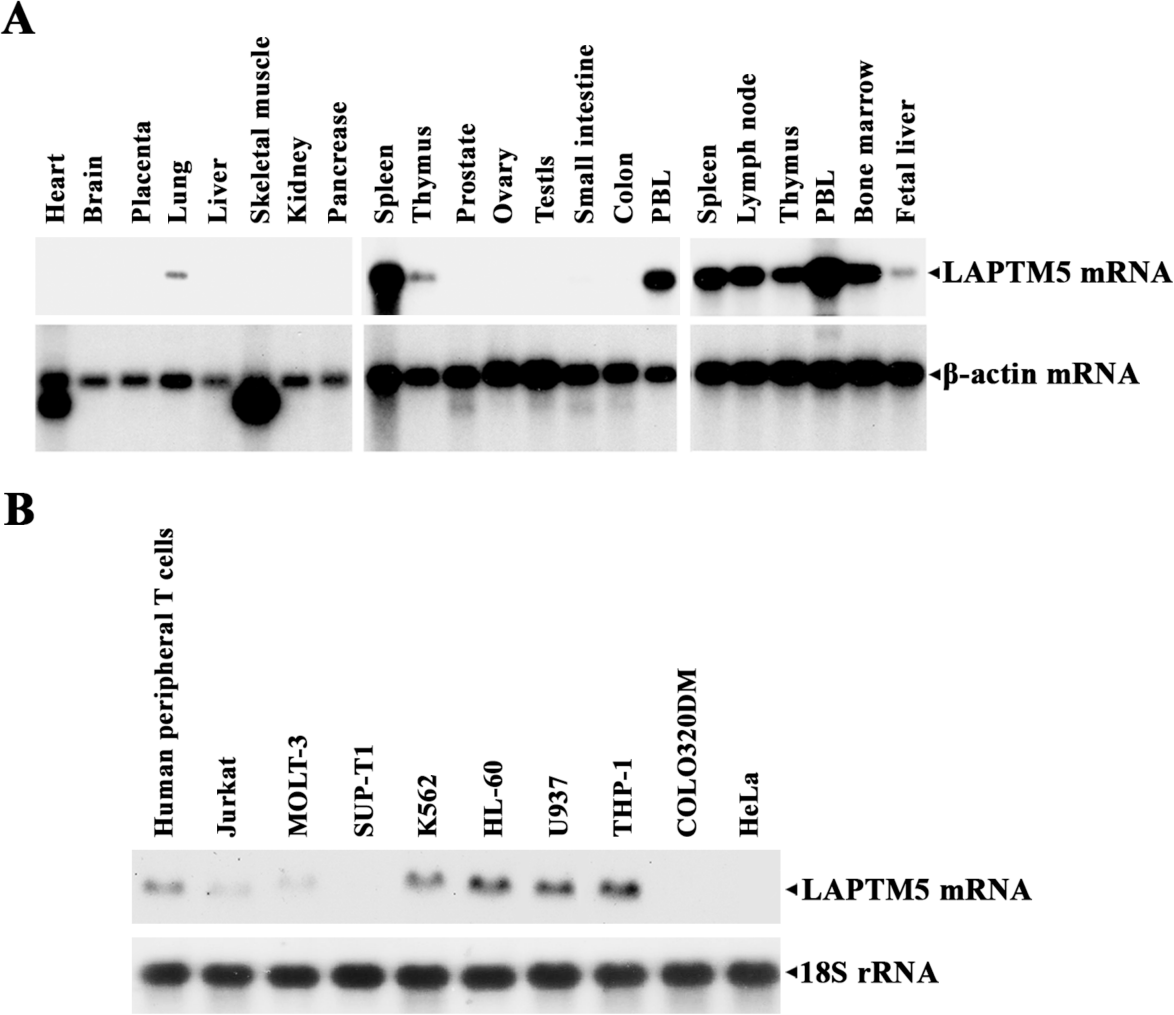
**Northern blot analyses of LAPTM5-specific mRNA in various human normal tissues (A) and in various human malignant cell lines (B).** Human multiple-tissue northern membranes and human immune system multiple-tissue northern membrane, each containing 2 μg of poly(A)^+^ RNA per lane, were sequentially hybridized using ^32^P-labeled human LAPTM5 and β-actin cDNA. The northern blot contained 10 μg of total RNA in each lane, which was isolated from each cell line, was hybridized with ^32^P-labeled human LAPTM5 and 18S rRNA cDNA.

Since LAPTM5 is preferentially expressed in hematopoietic cells [1], we investigated the expression of *LAPTM5* mRNA using human multiple-tissue northern blot panels I and II and human immune system multiple-tissue northern blot panel II, to confirm its cell and tissue distribution. As shown in **Fig 3A**, in healthy human tissues, *LAPTM5* mRNA was expressed at high levels in hematopoietic cells and tissues including peripheral blood lymphocytes (PBLs), the spleen, thymus, lymph node, and bone marrow with the most significant expression in the PBLs, and at low levels in the lung and fetal liver. However, there was no detectable *LAPTM5* mRNA in the heart, brain, placenta, liver, skeletal muscles, kidney, pancreas, prostate, testis, ovary, small intestine, and colon (mucosal lining). These results indicate that the expression of LAPTM5 is restricted to hematopoietic cells and tissues as well as the lung and fetal liver.

In order to determine whether the *LAPTM5* gene is differentially expressed in hematopoietic cells depending on myeloid- and lymphoid-lineage specificity, several malignant cells in both the myeloid and lymphoid lineage and non-hematopoietic malignant cells were analyzed for *LAPTM5* mRNA by northern blotting. As shown in **Fig 3B**, *LAPTM5* mRNA was detected in human peripheral T cells and immature malignant cells in the myeloid lineage such as chronic myelogenous leukemia K562 cells, promyelocytic leukemia HL-60 cells, promonocytic leukemia U937 cells, and acute monocytic leukemia THP-1 cells. *LAPTM5* mRNA was absent or hardly detected in the lymphoid malignant cells (acute T cell leukemia Jurkat cells, acute lymphoblastic leukemia Molt-3 cells, and T cell lymphoblastic lymphoma Sup-T1 cells) and non-hematopoietic malignant cells (colon adenocarcinoma COLO 320DM cells, and cervical epithelioid carcinoma HeLa cells). These results indicate that LAPTM5 expression was more dominant in hematopoietic cells in the myeloid lineage compared with those in the lymphoid lineage, and that *LAPTM5* mRNA was more abundant in resting mature hematopoietic cells than in their immature and/or continuously proliferating counterparts.

### Differential expression of LAPTM5 during lineage-specific terminal differentiation of HL-60 cells

**Fig 4:**
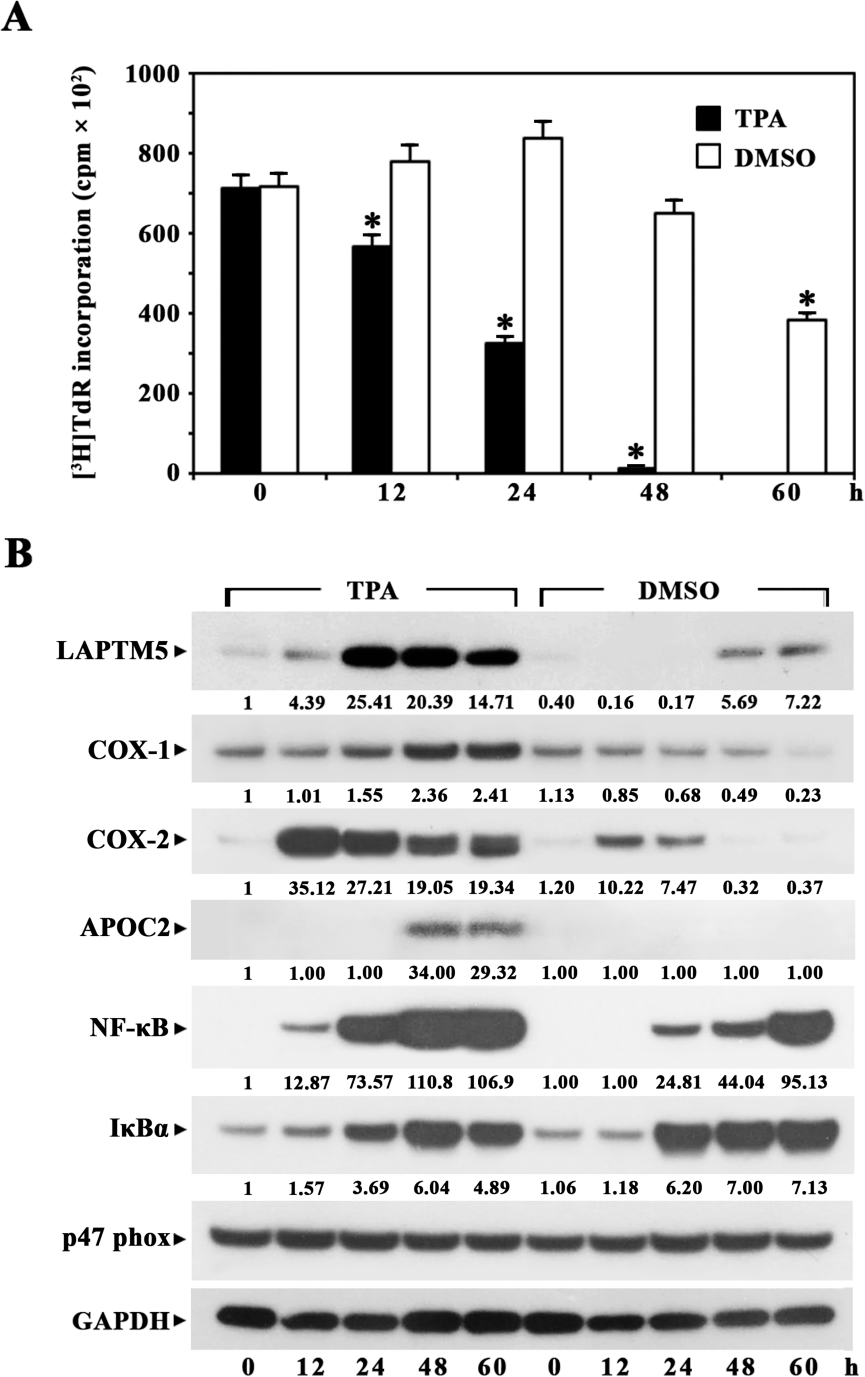
Kinetic analysis of [^3^H]TdR-incorporation (A) and western blot analysis of LAPTM5 protein (B) during TPA-induced differentiation of HL-60 cells into monocytes/macrophages or DMSO-induced differentiation of HL-60 cells into granulocytes. For the proliferation assay, HL-60 cells (1 × 10^5^ cells/well) were treated with 32 nM TPA or 1.25% DMSO in 96-well plates and pulsed for 4 h with 1 μCi of [^3^H]TdR at the times indicated. Equivalent cultures were incubated and the cells were harvested at the indicated times for preparation of cell lysate. Western blot analysis was performed as described in the Materials and Methods. Representative results are presented; two additional experiments yielded similar results.

Previously, it was reported that HL-60 cells differentiate into monocytes/macrophages in the presence of TPA or 1,25-dihydroxyvitamin D3, whereas the cells differentiate into granulocytes upon exposure to DMSO or retinoic acid [18, 27–29]. In this context, we induced terminal differentiation in HL-60 cells with TPA or DMSO, and investigated whether the expression profile of LAPTM5 protein during TPA-induced terminal differentiation of HL-60 cells into monocytes/macrophages differs from the expression profile during DMSO-induced terminal differentiation of HL-60 cells into granulocytes. As shown in **Fig 4A** and **4B**, cell growth-arrest occurred rapidly during 32 nM TPA-induced terminal differentiation of HL-60 cells and [^3^H]TdR incorporation was not detected after 48 h. The expression of LAPTM5 protein, which was detectable at a low level in HL-60 cells, increased ~25.4-fold at 24 h; this significantly enhanced level was sustained until 60 h after TPA-treatment. However, the rapid growth-arrest did not occur and the LAPTM5 protein level was enhanced ~7-fold during 1.25% DMSO-induced terminal differentiation of HL-60 cells into granulocytes. Additionally, the expression patterns of COX-1, COX-2, and NF-κB proteins were similar to that of the LAPTM5 protein, in that their levels were more significantly elevated during TPA-induced monocytic/macrophagic differentiation compared to the elevation during DMSO-induced granulocytic differentiation. In contrast, the IκBα protein level became more prominently up-regulated during DMSO-induced differentiation than during TPA-induced differentiation. Although the APOC2 protein was not detected in untreated HL-60 cells, it was markedly detected between 48 h and 60 h during TPA-induced differentiation, but not during DMSO-induced differentiation, as previously reported [15]. The expression level of p47phox, which was clearly detected in untreated HL-60 cells, remained relatively constant irrespective of treatment with TPA or DMSO.

These results indicate that although LAPTM5 protein was expressed at a low level in HL-60 cells, its levels increased significantly during terminal differentiation into monocytes/macrophages and that its expression might be more important for lysosomes in macrophages than in granulocytes.

### Ectopic expression of LAPTM5 in HeLa cells

**Fig 5:**
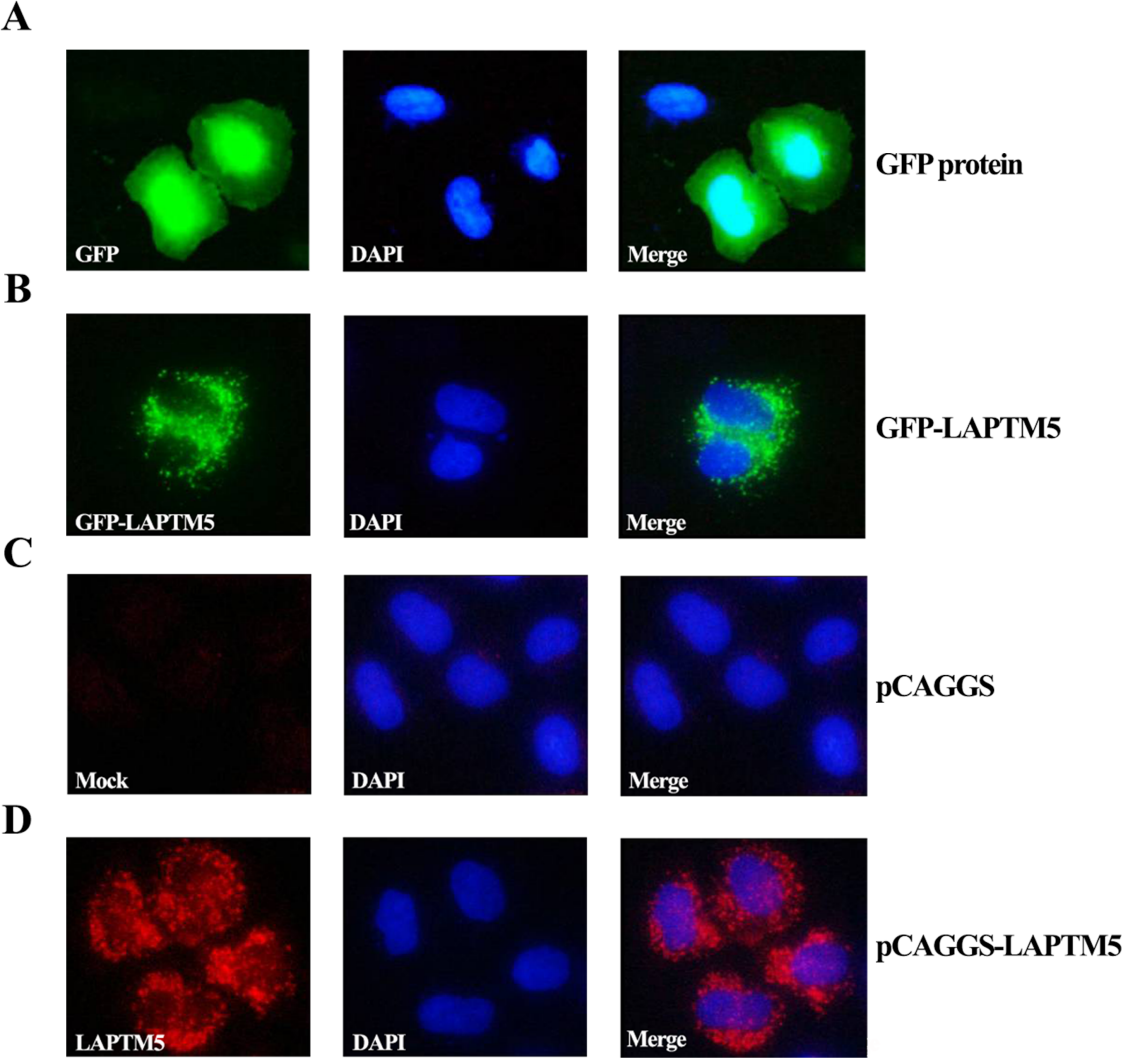
Localization of GFP (A) and GFP-LAPTM5 (B) in HeLa cells, and immunofluorescence staining by anti-LAPTM5 of HeLa cells transfected with pCAGGS (C) or pCAGGS-*LAPTM5* (D). To observe localization of GFP and GFP-LAPTM5, HeLa cells transfected with either a GFP vector or a GFP-*LAPTM5* vector were fixed with 4% paraformaldehyde for 30 min. For immunostaining of ectopically expressed LAPTM5 in HeLa cells, cells transfected with pCAGGS or pCAGGS-*LAPTM5* were fixed with cold methanol and stained with rabbit polyclonal anti-human LAPTM5 antibody and DAPI as described in the Materials and Methods. Images were visualized at ×200 using the LSM 700 confocal laser scanning microscope. Representative results are presented; two additional experiments yielded similar results.

To understand the functional role of LAPTM5, we examined the effect of ectopic expression of LAPTM5 in HeLa cells, which do not express a detectable level of *LAPTM5* mRNA. To confirm the subcellular localization of ectopically expressed LAPTM5 in HeLa cells, cells were transfected with a GFP-*LAPTM5* vector for 48 h, and the localization of GFP-LAPTM5 was visualized. As shown in **Fig 5A**, the GFP protein (control) was localized in both the nucleus and the cytoplasm. However, the GFP-LAPTM5 fusion protein was localized in the juxtanuclear region, consistent with the localization of LAPTM5 previously determined in hematopoietic cells [1], supporting its lysosomal localization (**Fig 5B**). In addition, we performed immunocytochemistry using the anti-LAPTM5 antibody in HeLa cells transfected with either pCAGGS or pCAGGS-*LAPTM5* to identify the subcellular localization of the LAPTM5 protein. While HeLa cells transfected with pCAGGS showed no staining (**Fig 5C**), the localization of LAPTM5 protein expressed by pCAGGS-*LAPTM5* was confined to the juxtanuclear region, similar to the localization of the GFP-LAPTM5 fusion protein (**Fig 5D**). This observation demonstrates that ectopic LAPTM5 was localized to the lysosome region of HeLa cells.

**Fig 6:**
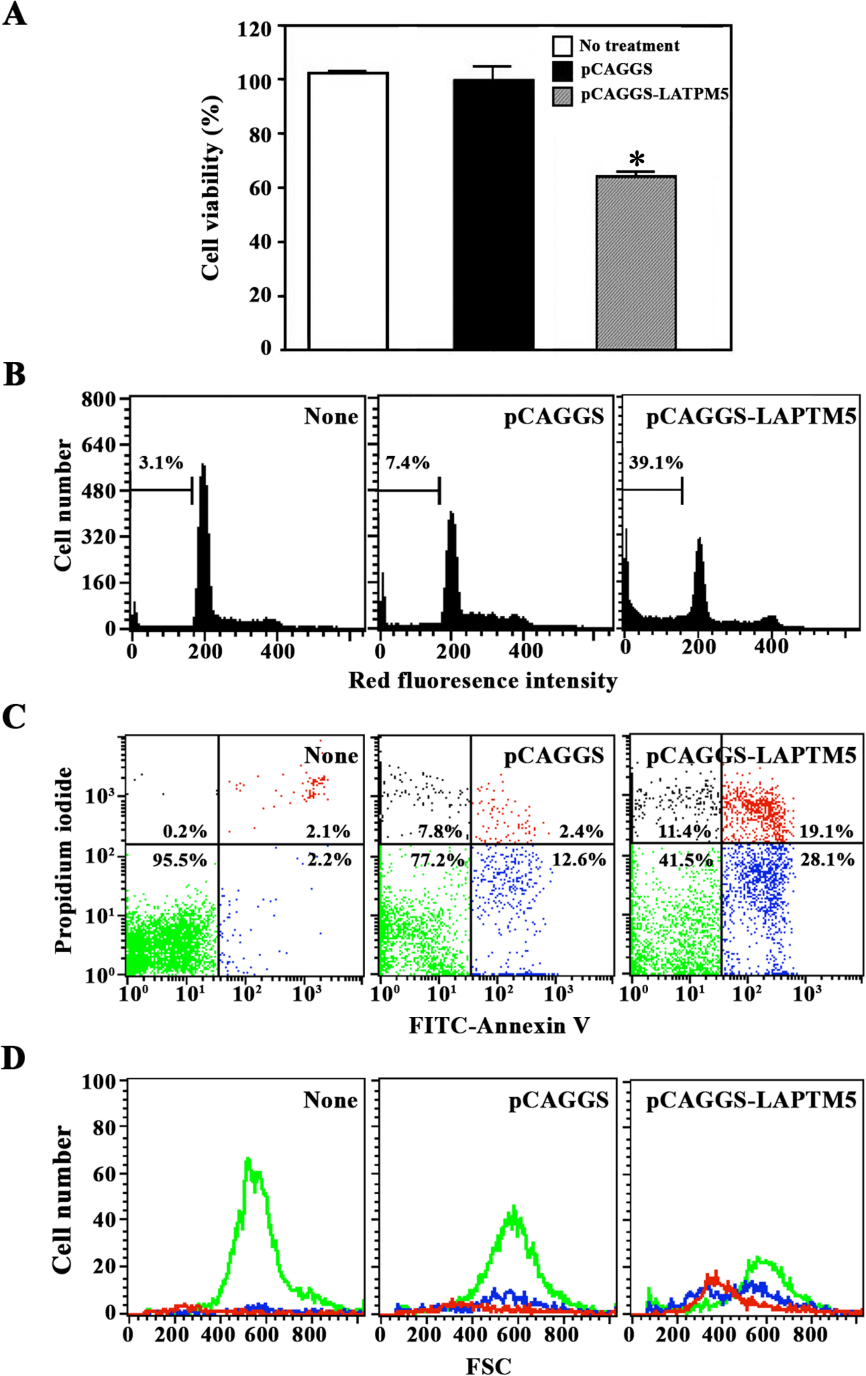
Effect of ectopic LAPTM5 expression on cell viability (A), cell cycle distribution (B), and apoptotic cell death measured by FITC-Annexin V and PI staining (C) and forward scatter dot plot (D) in HeLa cells. Untreated HeLa cells or those transfected with pCAGGS or pCAGGS-*LAPTM5* were harvested 48 h after transfection. Cell cycle distribution and apoptotic cell death were determined by flow cytometric analyses with PI staining and FITC-Annexin V/PI double staining, respectively. The forward scatter properties of individual unstained live, early apoptotic, and late apoptotic cells were measured to analyze changes in cell size during the induced apoptosis. For the cell viability assay, the individual cells harvested 12 h after transfection were added to a 96-well plate (1 × 10^4^ cells/well) and incubated for an additional 36 h, with MTT added for the final 4 h. The cells were sequentially processed to assess the colored formazan crystal produced from MTT as an index of cell viability. Each value is expressed as the mean ± SD (n = 3 with three replicates per independent experiment). *p < 0.05 compared to the control. Representative results are presented; two additional experiments yielded similar results.

To examine the involvement of LAPTM5 in cell proliferation and cell death, HeLa cells transfected with pCAGGS or pCAGGS-*LAPTM5* were harvested and their proliferation potentials were compared by MTT assay. Although the proliferation of HeLa cells transfected with pCAGGS was essentially the same as that of untreated HeLa cells, the proliferation of HeLa cells transfected with pCAGGS-*LAPTM5* decreased to 64% of the control (**Fig 6A**). Since the average transfection efficiency of HeLa cells with pCAGGS-*LAPTM5* gene was ~30% (data not shown), these results indicate that LAPTM5 in the lysosome was associated with suppression of cell proliferation.

Sequentially, to investigate whether the antiproliferative effect of ectopic LAPTM5 was attributable to cell cycle arrest and/or apoptotic cell death, cells transfected with pCAGGS or pCAGGS-*LAPTM5* were compared with the control HeLa cells by flow cytometric analysis following staining either with propidium iodide (PI) or with both FITC-Annexin V and PI. As shown in **Fig 6B**, although the proportion of the apoptotic sub-G_1_ peak in HeLa cells after transfection with pCAGGS appeared to be 7.4%, the proportion in HeLa cells increased to 39.1% after transfection with pCAGGS-*LAPTM5*. At the same time, there was no remarkable alteration in the percentage of cells in G_1_, S, or G_2_/M phase in HeLa cells following *LAPTM5* transfection. As shown in **Fig 6C**, HeLa cells transfected with pCAGGS-*LAPTM5* showed an increase in early apoptotic cells stained with only FITC-Annexin V, and late apoptotic cells stained with both FITC-Annexin V and PI. Under these conditions, however, the increase in necrotic cells stained with only PI was barely detected. Although the light scattering properties of the unstained live cells were not altered following *LAPTM5* transfection, the late and early apoptotic cells had a decrease in forward scatter, representing no cellular swelling but a reduction in cell size during LAPTM5-induced apoptosis (**Fig 6D**). These results indicate that ectopic LAPTM5 expression in HeLa cells was anti-proliferative by inducing apoptosis, not necrosis or cell cycle arrest.

**Fig 7:**
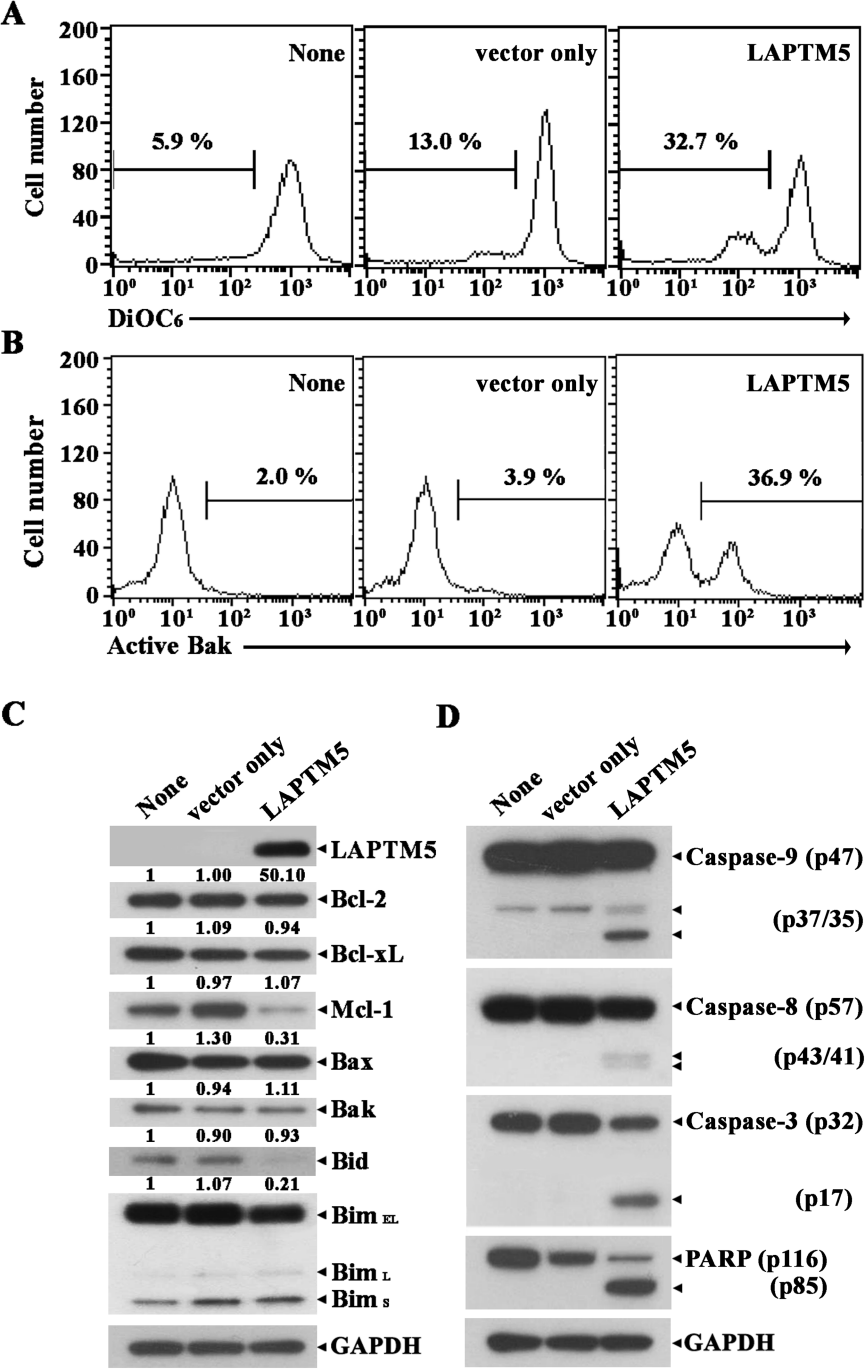
Flow cytometric analyses of Δψm loss (A), Bak activation (B), and western blot analyses of LAPTM5, Bcl-2, Bcl-xL, Mcl-1, Bak, Bax, Bid, Bim, and GAPDH (C), and activation of caspase-9, -8, and -3, PARP cleavage, and GAPDH (D) in HeLa cells ectopically expressing LAPTM5. HeLa cells untreated and transfected with pCAGGS or pCAGGS-*LAPTM5* were harvested 48 h after transfection. Flow cytometric analyses of Bak activation and Δψm loss, and western blot analyses were performed as described in the Materials and Methods. Representative results are presented; two additional experiments yielded similar results.

**Fig 8:**
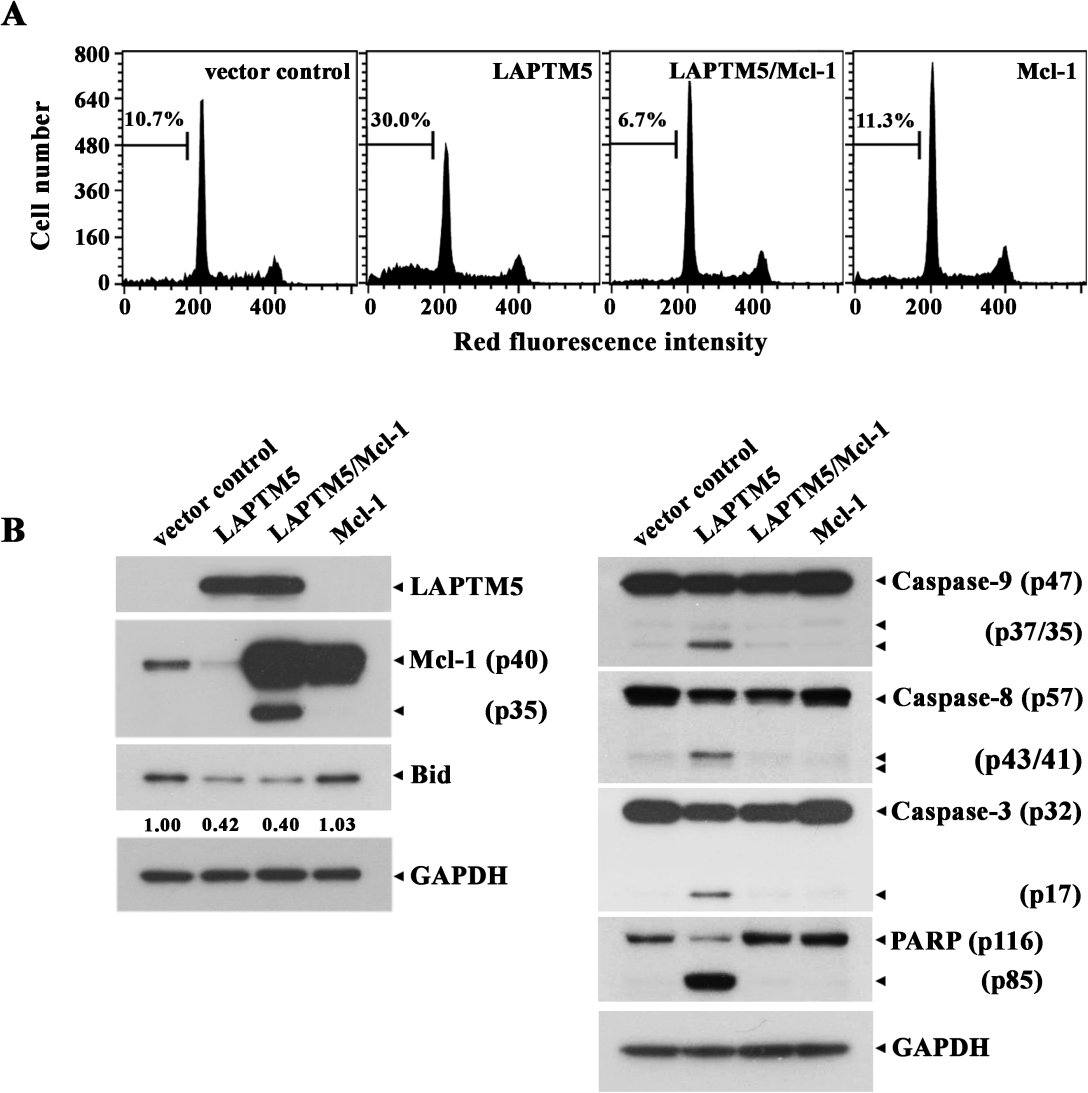
Effect of Mcl-1 overexpression on the ectopic LAPTM5-induced apoptotic sub-G_1_ peak (A), and changes in the level of pro-apoptotic and anti-apoptotic regulatory proteins (B) in HeLa cells. After HeLa cells were transfected with pCAGGS/pcDNA3.1, pCAGGS-*LAPTM5*/pcDNA3.1, pCAGGS-*LAPTM5*/pcDNA3.1-*Mcl-1*, or pCAGGS/pcDNA3.1-*Mcl-1* for 48 h, cells were harvested and subjected to cell cycle analysis and western blot analysis as described in the Materials and Methods. Representative results are presented; two additional experiments yielded similar results.

To elucidate the death-signaling pathway underlying LAPTM5-mediated apoptosis in HeLa cells, we measured the change in Δψm by flow cytometry using DiOC_6_ staining. Although the level of Δψm loss in HeLa cells transfected with pCAGGS was 9.9%, the Δψm loss was enhanced to a level of 32.7% following transfection with pCAGGS-*LAPTM5* (**Fig 7A**). The pro-apoptotic multidomain Bcl-2 family protein Bak has been shown to mediate permeabilization of the mitochondrial outer membrane, leading to mitochondrial cytochrome *c* release into the cytosol and resultant activation of the caspase cascade [30, 31]. To confirm that LAPTM5-mediated apoptotic cell death in HeLa cells is accompanied by Bak activation, we performed flow cytometry using the conformation-specific anti-Bak (Ab-1) to analyze the N-terminal conformational change required for Bak activation in the HeLa cells expressing LAPTM5. As shown in **Fig 7B**, Bak activation was detected in HeLa cells transfected with pCAGGS-*LAPTM5*, but not in HeLa cells transfected with pCAGGS. Additionally, western blot analysis showed that HeLa cells after transfection with pCAGGS-*LAPTM5* expressed ∼50-fold higher level of LAPTM5 protein compared to control HeLa cells transfected with pCAGGS, indicating ectopic overexpression of LAPTM5 (**Fig 7C**). In accordance with Δψm loss and Bak activation, an ~3.2-fold reduction in anti-apoptotic Mcl-1 protein level as well as caspase-9 activation that proceeded by proteolytic cleavage of the inactive proenzyme (47 kDa) to the active forms (37/35 kDa) was detected (**Fig 7D**). In addition, caspase-8 activation through proteolytic cleavage of the proenzyme (57 kDa) into the active forms (43/41 kDa), the cleavage of procaspase-3 (32 kDa) into the active form (17 kDa), and PARP degradation were detected in HeLa cells ectopically overexpressing LAPTM5. Under these conditions, the levels of anti-apoptotic Bcl-2 family members (Bcl-2 and Bcl-xL) and pro-apoptotic Bcl-2 family members (Bak, Bax, and Bim) remained relatively constant. The level of Bid protein (22 kDa), which is known to be cleaved by active caspase-8 [32, 33] or lysosomal cathepsins [34, 35] to generate truncated Bid (t-Bid, 15 kDa) causing Δψm loss and subsequent release of cytochrome *c*, appeared to decrease by an ~4.8-fold. However, the generation of tBid was not detected by western blot analysis in HeLa cells overexpressing LAPTM5, possibly attributable to the short half-life of tBid. To examine whether the decline of Mcl-1 level is crucial for provoking apoptotic cell death in HeLa cells overexpressing LAPTM5, we sought to determine the protective effect of concomitant overexpression of Mcl-1 on LAPTM5 overexpression-mediated apoptosis. As shown in **Fig 8A**, the LAPTM5-mediated apoptotic sub-G_1_ cells were completely abrogated by Mcl-1 overexpression. Simultaneously, the LAPTM5-mediated apoptotic events including activation of caspase-9, -8 and -3, and PARP degradation were blocked, whereas the decline in Bid level was not affected (**Fig 8B**). It is of interest to note that concomitant overexpression of LAPTM5 and Mcl-1 in HeLa cells produced a cleaved Mcl-1 (~35 kDa) along with the overexpressed Mcl-1 (40 kDa). However, although the level of endogenous Mcl-1 (40 kDa) was reduced by ~2.4-fold in HeLa cells overexpressing LAPTM5 alone, the cleaved Mcl-1 was not detected presumably due to its relatively low abundance. These results indicate that the decline of Mcl-1 level was a prerequisite for LAPTM5 overexpression-mediated apoptotic cell death in HeLa cells, and suggest that the decline of both Mcl-1 and Bid levels occurred via their cleavage by the lysosomal pathway, but not by the mitochondrial caspase cascade pathway.

**Fig 9:**
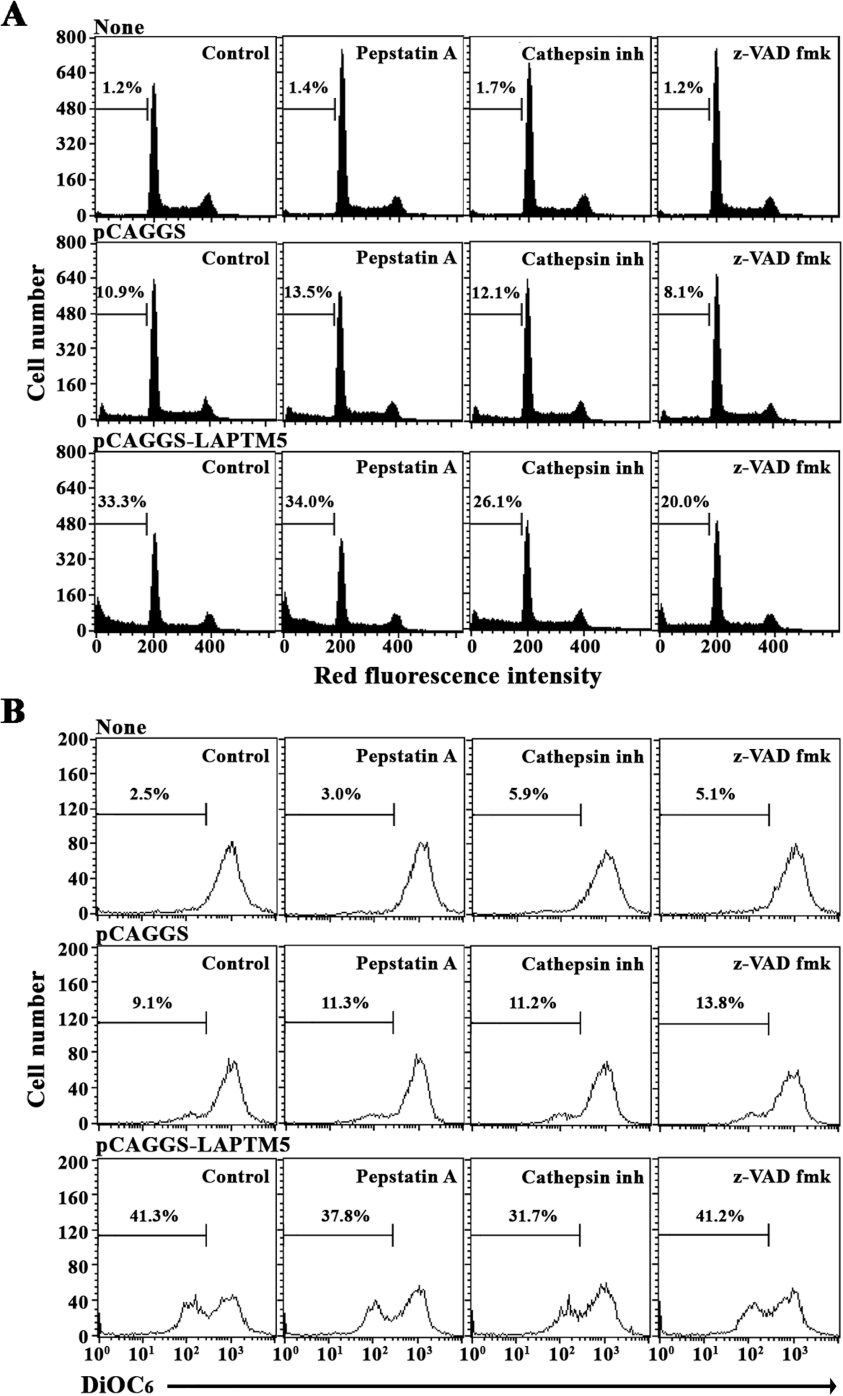
Effect of the pan-caspase inhibitor (z-VAD-fmk), the pan-cathepsin inhibitor (Cathepsin inhibitor I), and the cathepsin D inhibitor (Pepstatin A) on the ectopic LAPTM5 overexpression-induced apoptotic sub-G_1_ peak (A) and Δψm loss (B) in HeLa cells. After HeLa cells were transfected with pCAGGS or pCAGGS-LAPTM5 for 12 h, 30 μM z-VAD-fmk, 2.5 μM Cathepsin inhibitor I, or 1.0 μM Pepstatin A were added to the individual cells and incubated for an additional 36 h. The apoptotic sub-G_1_ peak and Δψm loss of the cells were analyzed using flow cytometry as described in the Materials and Methods. Representative results are presented; two additional experiments yielded similar results.

To examine further the involvement of cathepsins or caspases in the LAPTM5 overexpression- mediated apoptosis in HeLa cells, the effects of the cathepsin D inhibitor (Pepstatin A), the pan-cathepsin inhibitor (Cathepsin inhibitor I), or the pan-caspase inhibitor (z-VAD-fmk) on the level of apoptotic sub-G_1_ peak and Δψm loss were investigated in HeLa cells transfected with pCAGGS-*LAPTM5*. As shown in **Fig 9A** and **9B**, while either Cathepsin inhibitor I or z-VAD-fmk could suppress the LAPTM5-mediated apoptotic sub-G_1_ peak, only Cathepsin inhibitor I, not z-VAD-fmk, appeared to reduce the LAPTM5-mediated Δψm loss, suggesting that the LAPTM5-mediated Δψm loss was exerted in a cathepsin-dependent manner. Under these conditions, neither the LAPTM5-mediated apoptotic sub-G_1_ peak nor Δψm loss was markedly influenced by the cathepsin D inhibitor (Pepstatin A), excluding the involvement of cathepsin D in the LAPTM5 overexpression-mediated apoptosis in HeLa cells.

Consequently, these results demonstrate that ectopic overexpression of LAPTM5 in HeLa cells induced apoptotic cell death, without accompanying necrosis, via cathepsin(s)-dependent cleavage of Mcl-1 and Bid, which rendered the cells susceptible to the onset of Bak activation and Δψm loss, with resultant activation of caspase-9, -8, and -3, leading to PARP degradation. Additionally, these results suggest that the caspase 8-mediated cleavage of Bid into tBid, if any, occurred as a down-stream event of mitochondrial damage rather than an initial event that causes mitochondrial damage.

## Discussion

This is the first report to demonstrate that ectopic expression of LAPTM5 in HeLa cells causes cathepsin-dependent cleavage of Mcl-1 and Bid, which subsequently provokes mitochondrial pathway of apoptosis via Bak activation, Δψm loss, and activation of caspase-9, -8, and -3, leading to PARP degradation. Flow cytometric analysis of the cells after staining with FITC-Annexin V and PI showed no remarkable necrosis during LAPTM5 overexpression-mediated apoptosis. The human *LAPTM5* gene was identified in this study by ODD-PCR as an up-regulated transcript during TPA-induced terminal differentiation of U937 cells into monocytes/macrophages.

Previous studies show that LAPTM5 is preferentially expressed in adult hematopoietic cells and localizes to lysosomal membranes [1]. We further investigated whether *LAPTM5* mRNA expression is restricted to hematopoietic cells. Additionally, we sought to identify any differential expression patterns between lymphoid and myeloid lineages of hematopoietic cells. To analyze *LAPTM5* mRNA expression, we performed a human multiple-tissue northern blot and a northern blot using total RNA isolated from lymphoid lineage-malignant cells, myeloid lineage-malignant cells, and non-hematopoietic malignant cells. In healthy human tissues, *LAPTM5* mRNA was mainly expressed at high levels in hematopoietic cells and tissues, such as PBLs, the spleen, thymus, lymph node, and bone marrow, with a maximum expression in PBLs. Low levels of *LAPTM5* mRNA were detected in the lung and fetal liver. Additionally, the expression of *LAPTM5* mRNA was more dominant in the myeloid lineage cells (K562, HL-60, U937, and THP-1) compared to the lymphoid lineage cells (Jurkat, Molt-3, and Sup-T1). The level of *LAPTM5* mRNA in unstimulated peripheral T cells was higher than that in immortalized malignant T cell lines. These results indicate that *LAPTM5* gene expression was more abundant in mature resting hematopoietic cells than in immature and/or continuously proliferating hematopoietic cells. The level of *LAPTM5* mRNA in unstimulated peripheral T cells declined after activation with anti-CD3 and anti-CD28 antibodies (data not shown). The higher expression of *LAPTM5* gene in unstimulated T cells compared with activated T cells is compatible with the previously proposed function of LAPTM5 in T cells; namely, that—LAPTM5 is associated with lysosomal degradation of the T cell receptor and thus down-regulation of the T cell activation response [8].

The LAPTM5 protein level significantly increased during TPA-induced differentiation of promyelocytic HL-60 cells into monocytes/macrophages; however, it increased to a lesser extent during DMSO-induced differentiation of HL-60 cells into granulocytes. Although the functional role of LAPTM5 in neutrophils remains unknown, it has recently been reported that LAPTM5 augments Toll-like receptor (TLR)-inflammatory signaling pathways and the secretion of proinflammatory cytokines in response to TLR ligands, as a critical role in macrophages [5]. Since the granulocytes derived from DMSO-induced differentiation of HL-60 cells are known to display neutrophil-like functional characteristics [28, 29, 36], and since neutrophils are similar to macrophages in their phagocytic and proinflammatory actions [37], current results revealing less extensive expression of LAPTM5 in DMSO-treated HL-60 cells compared to TPA-treated cells suggest that the LAPTM5-associated lysosomal function required for the TLR-mediated proinflammatory response might be more prominent in macrophages than in neutrophils.

While several studies have recently focused on the functional role of LAPTM5 in hematopoietic cells, such as macrophages and T and B lymphocytes [5, 8, 9], we sought to examine the role of LAPTM5 in non-hematopoietic cells. We investigated the effect of ectopic expression of LAPTM5 in HeLa cells, in which both *LAPTM5* mRNA and LAPTM5 protein were not detected by northern and western blot analyses, respectively. In our experimental model, the ectopically expressed LAPTM5 protein in HeLa cells was translocated to the lysosome as evidenced by the intracellular localization of GFP-LAPTM5 and immunostaining results using an anti-LAPTM5 antibody. Following pCAGGS-*LAPTM5* transfection, the viability of HeLa cells overexpressing LAPTM5 protein declined to ~64% of the viability of mock-transfected cells. In addition, the levels of Δψm loss and early and late apoptotic cells stained with FITC-Annexin V and with FITC-Annexin V and PI, respectively, were elevated. This suggested that apoptotic cell death, without accompanying necrosis, was induced in HeLa cells following LAPTM5 overexpression. LAPTM5 accumulation in HeLa cells following pCAGGS-*LAPTM5* transfection was accompanied by reduction of Mcl-1 and Bid levels, Bak activation, Δψm loss, and activation of caspase-9, -3, and -8, thereby leading to PARP degradation, demonstrating that overexpressed LAPTM5 is involved in the lysosomal contribution to activation of a mitochondria-dependent apoptotic pathway in HeLa cells.

Previous studies have reported that altering the expression ratio of pro-apoptotic Bcl-2 family members (Bad, Bak, Bax, Bid, Bim, and Puma) to anti-apoptotic Bcl-2 family members (Bcl-2, Bcl-xL, and Mcl-1) is associated with the activation of Bak and/or Bax during mitochondria damage-mediated apoptosis induced by chemotherapeutic agents [38, 39]. Although the levels of Bak, Bax, Bim, Bcl-2, and Bcl-xL remained relatively constant in HeLa cells ectopically overexpressing LAPTM5, there was a significant reduction in the level of Mcl-1. At the same time, the level of Bid (22 kDa), which can be cleaved by active caspase-8 to generate tBid (15 kDa) causing Δψm loss [33], was significantly reduced in accordance with caspase-8 activation. Since caspase-8 was often activated downstream of caspase-3 to comprise a positive feedback loop involving tBid-mediated mitochondrial cytochrome *c* release in chemical agent-induced apoptosis of tumor cells [40–42], current data could not exclude the possibility that the caspase-8 activation and Bid cleavage observed in HeLa cells overexpressing LAPTM5 were due to the mitochondrial damage-mediated activation of caspase-9 and -3. Previously, Blomgran et al. have reported that ROS-mediated lysosomal membrane permeabilization (LMP) in neutrophils causes apoptosis via cathepsin-mediated cleavage of Mcl-1 and Bid, and resultant mitochondrial damage and caspase activation [34]. It has also been reported that the accumulation of LAPTM5 protein in Ad-*LAPTM5*-infected neuroblastoma cells induces caspase-independent non-apoptotic cell death with lysosomal destabilization and LMP, which allows lysosomal cathepsin D release into the cytosol [13]. In our experiment, the LAPTM5 overexpression-induced apoptotic sub-G_1_ peak in HeLa cells was reduced by ~40% in the presence of the pan-caspase inhibitor (z-VAD-fmk); however, the induced Δψm loss was not affected, indicating that the mitochondrial damage and subsequent activation of the caspase cascade were associated with the LAPTM5-induced apoptotic cell death. Additionally, the pan-cathepsin inhibitor (Cathepsin inhibitor I) could suppress the LAPTM5-induced apoptotic sub-G_1_ peak and Δψm loss by ~22% and ~23%, respectively, implying the contribution of cathepsin(s) to the Δψm loss and apoptotic cell death. However, neither the LAPTM5-induced apoptotic sub-G_1_ peak nor Δψm loss was prominently influenced by the cathepsin D inhibitor (Pepstatin A), excluding the involvement of cathepsin D in LAPTM5 overexpression-induced apoptosis in HeLa cells.

Recently, it has been reported that HeLa cells are sensitive to lysosomotropic drug-mediated lysosomal disruption, which results in apoptosis induction via Bid cleavage by lysosomal cathepsins [43]. More recently, it has also been shown that LAPTM5 overexpression in human esophageal squamous cell carcinoma KYSE170 cells is able to promote release of cathepsins from lysosomes and cell death [44]. These previous data are consistent with our findings in this paper, in that LAPTM5 overexpression in HeLa cells can lead to release of lysosomal cathepsin(s), which causes the cleavage of Mcl-1 and Bid, and resultant mitochondrial apoptotic cell death. However, it remains to be elucidated whether and which isoforms of lysosomal cathepsins are responsible for the cleavage of Mcl-1 and Bid, thus reducing their levels, in HeLa cells ectopically overexpressing LAPTM5.

In conclusion, both mRNA and protein levels of LAPTM5 were significantly up-regulated during TPA-induced differentiation of U937 into monocytes/macrophages. While the LAPTM5 protein level increased during TPA-induced differentiation of HL-60 cells into monocytes/macrophages, the increase was lower during DMSO-induced differentiation of HL-60 cells into granulocytes. In healthy human tissues, the expression of *LAPTM5* mRNA was mainly detected in hematopoietic cells and tissues as well as the lung and fetal liver; however, it was not detected in other non-hematopoietic tissues. Ectopic overexpression of LAPTM5 in HeLa cells resulted in the localization of LAPTM5 protein to the lysosome. Additionally, cell viability declined largely due to mitochondria-dependent apoptosis resulting from cleavage of Mcl-1 and Bid, Bak activation, Δψm loss, and activation of caspase-9, -8, and -3, leading to PARP degradation. These results suggest that the up-regulation of LAPTM5 expression in cervical cancer may be a promising strategy for development of novel anticancer therapies.

## Supporting information

S1 Fig ODD-PCR images for different 3'-cDNA fragments compared in three samples. Total RNAs from untreated U937 cells and 32 nM TPA-treated (for 18 h or 48 h) U937 cells were reverse transcribed, and sequentially the obtained 3'-end *Rsa*I-restriction fragments of cDNAs were amplified by PCR as described in the Materials and Methods. The PCR products were electrophoresed on a 6% polyacrylamide sequencing gel and detection of the amplified cDNA fragments was visualized by autoradiography after the dried gel was exposed to X-ray film.
(TIF)
