# Supplementary figures and images for: Ectopic overexpression of LAPTM5 results in lysosomal targeting and induces Mcl-1 down-regulation, Bak activation, and mitochondria-dependent apoptosis in human HeLa cells
Source: PLoS One. 2017 May 2;12(5):e0176544. doi: 10.1371/journal.pone.0176544 (PMC5413007; doi:10.1371/journal.pone.0176544)

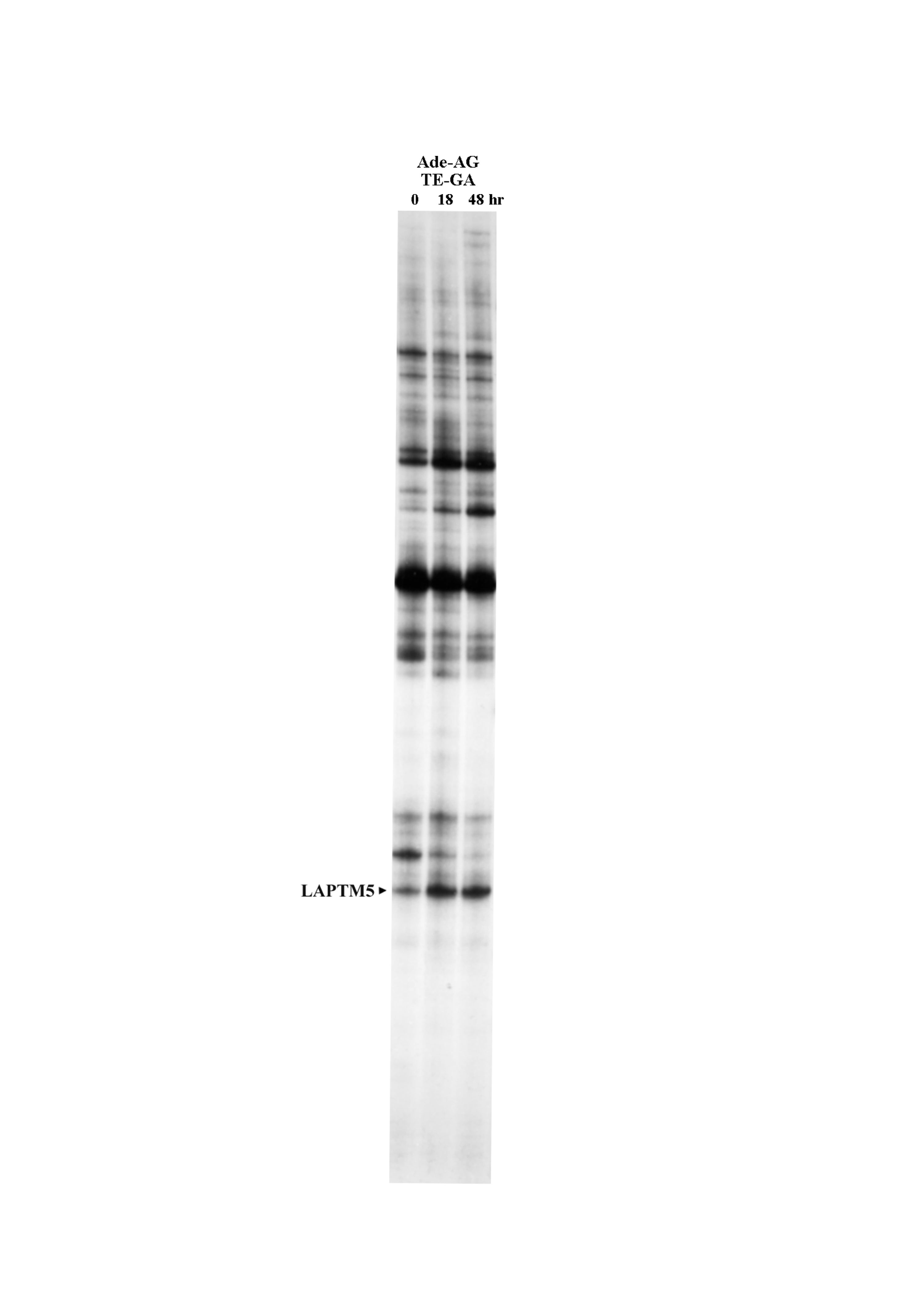

Supplement: S1 Fig — Total RNAs from untreated U937 cells and 32 nM TPA-treated (for 18 h or 48 h) U937 cells were reverse transcribed, and sequentially the obtained 3'-end RsaI-restriction fragments of cDNAs were amplified by PCR as described in the Materials and Methods. The PCR products were electrophoresed on a 6% polyacrylamide sequencing gel and detection of the amplified cDNA fragments was visualized by autoradiography after the dried gel was exposed to X-ray film. (TIF) [file pone.0176544.s001.tif]
